# Cognitive training enhances growth mindset in children through plasticity of cortico-striatal circuits

**DOI:** 10.1038/s41539-022-00146-7

**Published:** 2022-11-12

**Authors:** Lang Chen, Hyesang Chang, Jeremy Rudoler, Eydis Arnardottir, Yuan Zhang, Carlo de los Angeles, Vinod Menon

**Affiliations:** 1 Department of Psychology, Santa Clara University, Santa Clara, CA 95053 USA; 2 Neuroscience Program, Santa Clara University, Santa Clara, CA 95053 USA; 3 Department of Psychiatry and Behavioral Sciences, Stanford University, Stanford, CA 94305 USA; 4 Department of Neurology and Neurological Sciences, Stanford University, Stanford, CA 94305 USA; 5 Stanford Neuroscience Institute, Stanford University, Stanford, CA 94305 USA

**Keywords:** Human behaviour, Motivation, Motivation, Neural circuits, Problem solving

## Abstract

Growth mindset, the belief that one’s abilities can improve through cognitive effort, is an important psychological construct with broad implications for enabling children to reach their highest potential. However, surprisingly little is known about malleability of growth mindset in response to cognitive interventions in children and its neurobiological underpinnings. Here we address critical gaps in our knowledge by investigating behavioral and brain changes in growth mindset associated with a four-week training program designed to enhance foundational, academically relevant, cognitive skills in 7–10-year-old children. Cognitive training significantly enhanced children’s growth mindset. Cross-lagged panel analysis of longitudinal pre- and post-training data revealed that growth mindset prior to training predicted cognitive abilities after training, providing support for the positive role of growth mindset in fostering academic achievement. We then examined training-induced changes in brain response and connectivity associated with problem solving in relation to changes in growth mindset. Children’s gains in growth mindset were associated with increased neural response and functional connectivity of the dorsal anterior cingulate cortex, striatum, and hippocampus, brain regions crucial for cognitive control, motivation, and memory. Plasticity of cortico-striatal circuitry emerged as the strongest predictor of growth mindset gains. Taken together, our study demonstrates that children’s growth mindset can be enhanced by cognitive training, and elucidates the potential neurobiological mechanisms underlying its malleability. Findings provide important insights into effective interventions that simultaneously promote growth mindset and learning during the early stages of cognitive development.

## Introduction

Growth mindset, the belief in improvements in one’s cognitive abilities through personal effort, is a psychological construct with broad implications for enabling students to reach their highest potential^1,2^. Endorsement of growth mindset has been linked to higher academic achievement and long-term professional outcomes^3,4^. Despite decades of behavioral research, systematic investigations of changes in growth mindset in children’s response to training have, however, been lacking. Moreover, the neurocognitive systems that support changes in growth mindset in response to training in children are not known. Addressing these knowledge gaps has the potential to provide important insights into the inter-relations between growth mindset and learning during the early stages of cognitive development. Here, we investigate whether a training focused on enhancing foundational cognitive skills in children can also change growth mindset and identify specific brain systems that underlie gains in growth mindset in response to cognitive training.

In the past two decades, there has been a growing interest in interventions that enhance growth mindset as a way to promote students’ learning (for a review, see ref. ^5^). Interventions involving a brief lesson on neural plasticity, which informs individuals that the brain is malleable with learning, have reported some promising findings^6^. For example, a two-session online growth mindset intervention in a large sample (*N* = 12,490) of high school students led to significant improvements in grade point average and increased enrollment in advanced mathematics courses^7^. However, findings have not been consistent across studies^8,9^, possibly due to individual differences in the effectiveness of growth mindset interventions^10^. Notably, a meta-analysis of 43 studies found that the effects of growth mindset interventions on academic achievement are modest, with an effect size (Cohen’s *d*) of 0.08 for the mean difference between intervention and control groups^9^. A broader unresolved issue is whether cognitive training could lead to changes in growth mindset. Academic interventions in children and adolescents increase positive attitude, which may be indicative of changes in growth mindset^11–13^. In late middle-aged and older adults, a multi-skill learning intervention was found to result in increased growth mindset^14^. However, there have been no direct investigations of cognitive training-related changes in growth mindset in children. In addition, the role of growth mindset in individual differences in response to cognitive training remains unclear, as both positive^14–16^ and negative^17,18^ as well as non-significant^19,20^ relations between growth mindset and training-related performance gains have been reported.

Crucially, to the best of our knowledge, although neural plasticity is often implied in growth mindset interventions, whether enhancements of growth mindset are associated with brain plasticity in children has not been directly examined. Extant cross-sectional EEG and fMRI studies have pointed to several brain systems that may be associated with growth mindset, including those that support cognitive control, motivation, and learning and memory. For example, studies using event-related potentials have revealed enhanced error monitoring signals, most likely originating from the anterior cingulate cortex (ACC)^21^, in individuals who endorse higher growth mindset^22–24^. Growth mindset has also been associated with the striatum, a basal ganglia region crucial for motivation and reward-related learning^25,26^. One resting-state fMRI study found that growth mindset was correlated with functional connectivity between the striatum and dorsal ACC and dorsolateral prefrontal cortex^27^. In addition, the hippocampal learning and memory system, which has been linked to positive attitude to math^28^, may also facilitate growth mindset. Taken together, while extant studies hint at putative brain systems underlying growth mindset, the neurobiological mechanisms underlying changes in growth mindset associated with cognitive training remain unknown.

**Fig. 1:**
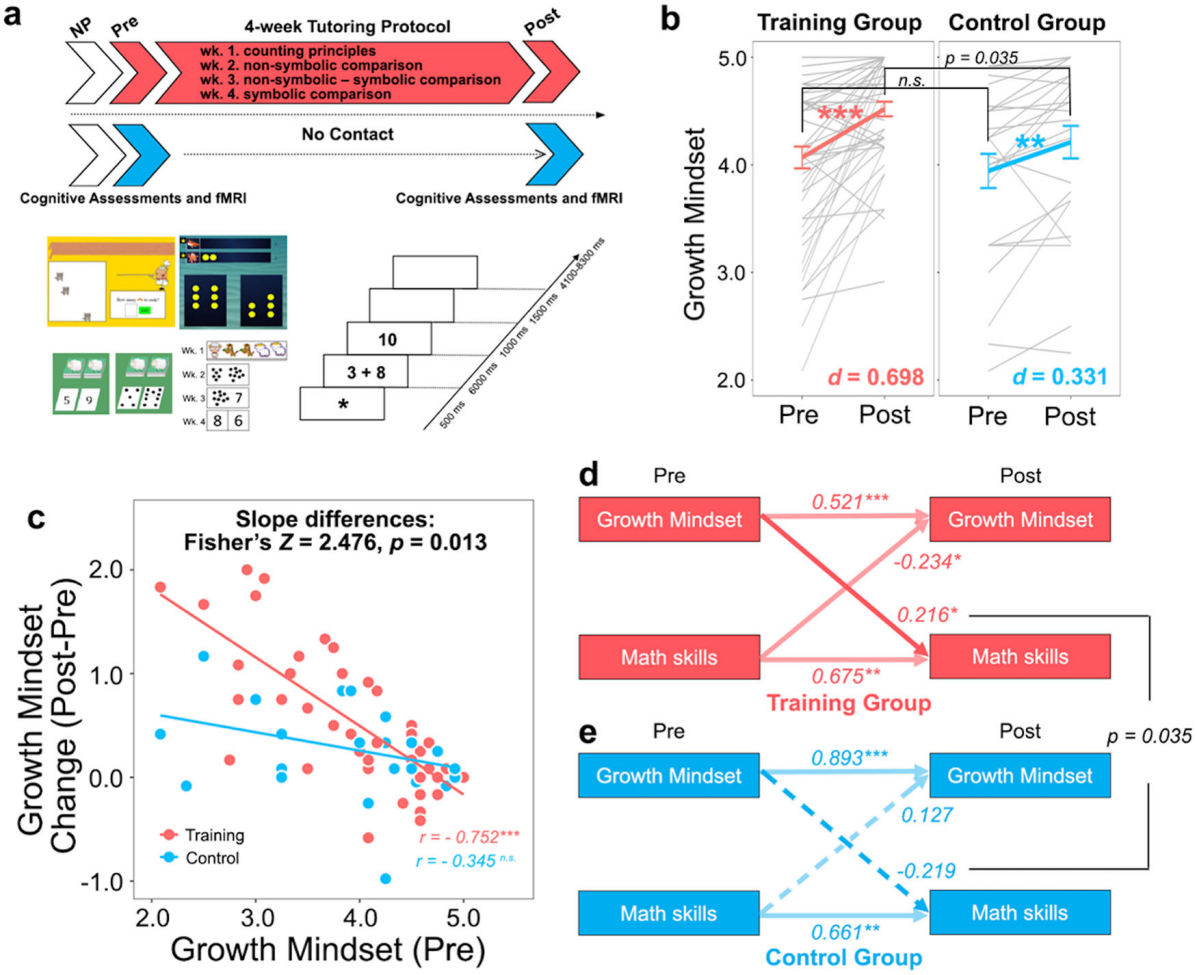
Overview of study design and training-related changes in growth mindset in children. **a** The study included multiple visits, with neuropsychological assessments (NP) at the beginning of the study and cognitive assessments and fMRI sessions at pre- (Pre) and post- (Post) visits. Children in the training group participated in a 4-week tutoring-based training program and those in the control group completed all parts of the study except for training. Sample tutoring materials and fMRI task are shown. Additional description of the tutoring protocol and fMRI task design can be found in *Methods*. Adapted from Chang et al.^29^. **b** Greater endorsement of growth mindset was observed in the training, compared to control, group at post-visit after the 4-week period. Group means are shown in thicker lines and individual trajectories are shown in thinner gray lines. Error bar shows standard error of mean. **c** Growth mindset prior to training correlates with changes in growth mindset in the training (red) group, with a significant difference in slope between training and control (blue) groups. **d**, **e** Cross-lagged panel analysis in structural equation modeling (SEM) shows a significantly greater cross-lagged effect of growth mindset on math skills (WJ-III Math Fluency) in the (**d**) training (solid red line), compared to (**e**) control (dotted blue line), group. Values represent standardized path estimates. *p < .05, **p < .01, ***p < .001, *n.s*. not significant.

Here we investigate changes in growth mindset, and their neural basis, associated with a unique tutoring-based training program designed to enhance foundational cognitive skills in early elementary school children (ages 7–10 years). Critically, our individualized training program (see *Methods* and Fig. 1a for details) uniquely focused on children’s learning of cognitive skills rather than exclusively instructing the concept of growth mindset. Although targeted growth mindset interventions have been shown to be effective, it remains to be determined whether children’s positive learning experiences can also enhance growth mindset. We reasoned that our training program encouraged children to focus on learning to improve their skills, rather than to demonstrate their ability, and therefore, was well-aligned to the core concept of growth mindset^5^. We first examined changes in children’s growth mindset across 4 weeks of training, compared to business-as-usual schooling, to determine whether the cognitive training program enhanced growth mindset. Based on previous studies suggesting that academic interventions not only improve academic performance but also change students’ attitudes by fostering positive learning experiences^11,13^, we hypothesized that children in the training, compared to control, group would show higher growth mindset in response to training. Next, we used structural equation modeling to test the hypothesis that higher levels of growth mindset prior to training would lead to better cognitive performance with training. Finally, we used task-based fMRI to examine the neural basis of changes in growth mindset in children in response to cognitive training. Based on cross-sectional studies on neural correlates of growth mindset or positive academic attitudes in children^23,27,28^, we predicted that neural plasticity in cognitive control, motivational, and memory systems would support growth mindset gains.

## Results

### Changes in children’s growth mindset after 4 weeks of cognitive training

The first goal of our study was to examine whether children who received 4 weeks of cognitive training showed higher levels of growth mindset relative to business-as-usual control group after the same time interval. In a mixed ANCOVA (see *Methods*), we found significant main effects of group (training, control) and time (pre-, post-training) (*F*s > 4.975, *p*s < 0.029, *η*^2^s > 0.032) and a significant group by time interaction, *F*(1,76) = 4.975, *p* = 0.029, *η*^2^ = 0.032 (Fig. 1b). Follow-up two-tailed *t*-tests confirmed that children’s growth mindset increased significantly in both training, *t*(51) = 5.052, *p* < 0.001, Cohen’s *d* = 0.698, 95% CI [0.392, 1.003], and control, *t*(26) = 3.287, *p* = 0.003, Cohen’s *d* = 0.331, 95% CI [0.123, 0.538], groups. While growth mindset was not significantly different between training and control groups prior to training, *t*(77) = 0.695, *p* = 0.489, Cohen’s *d* = 0.164, 95% CI [−0.308, 0.638], this group difference was significant after 4 weeks, *t*(77) = 2.142, *p* = 0.035, Cohen’s *d* = 0.508, 95% CI [0.029, 0.987], with higher levels of growth mindset in the training group (*M* = 4.52, SD = 0.49), compared to the controls (*M* = 4.21, SD = 0.79). Given that the two groups displayed the same level of growth mindset prior to training, it is less likely that higher levels of growth mindset at post-test in the training group are associated with “more room to improve.” Together, these results demonstrate that children who received cognitive training showed greater increases in growth mindset than those who did not participate in the cognitive training program.

### Relation between growth mindset prior to training and gains in growth mindset after training

Next, we examined how training-related changes in growth mindset were associated with individual differences in growth mindset prior to training. A careful examination of our data indicated that most of participants across both groups (72.2%) showed positive changes in their growth mindset over 4 weeks, including those with above average levels of growth mindset at pre-visit (Fig. 1c). Our analysis revealed that individual differences in the degree of improvements of growth mindset scores were strongly negatively correlated with growth mindset scores prior to training in the training group, *r*(50) = −0.752, *p* < 0.001, Cohen’s *d* = 2.280, 95% CI [1.448, 3.113]: those with lower levels of growth mindset prior to training showed greater gains in growth mindset. More importantly, this association was significantly different from that in the control group, *Z* = 2.476, *p* = 0.013 (Fig. 1c; Supplementary Tables 4–5), and this association did not reach significance in the control group, *r*(25) = −0.345, *p* = 0.078, Cohen’s *d* = 0.736, 95% CI [−0.083, 1.555]. Our results show that (i) most children, including those with above average levels of growth mindset at pre-visit, showed positive gains in their growth mindset, and (ii) those with lower levels of growth mindset prior to training had higher gains in growth mindset, with a stronger association in the training, compared to control, group. These two observations suggest that our findings cannot be simply atrributed to “regression to the mean” and reveal systematic training-induced changes in growth mindset explained by growth mindset prior to training across individuals.

### Influence of growth mindset on math skills in response to cognitive training

We next implemented a cross-lagged panel analysis using structural equation modeling (SEM) to test whether growth mindset prior to training predicts post-training math skills measured by standardized scores from the Math Fluency subtest of the WJ-III in the training, compared to control, group. We found that growth mindset prior to training was associated with post-training math skills in the training group, *β* = 0.216, *p* = 0.025, which suggests that higher levels of growth mindset lead to better math performance with training (Fig. 1d). This relationship was not observed in the control group, *β* = −0.219, *p* = 0.191 (Fig. 1e). A comparison between baseline (unconstrained) and constrained models in the multi-group analysis revealed that the constrained model fit worse than the baseline model, *χ*^2^(1) = 4.433, *p* = 0.035, suggesting that the predictive role of growth mindset at pre-training on post-training math skills was significantly different between training and control groups (Supplementary Table 6). Additionally, a negative relationship between math skills prior to training and post-training growth mindset was observed in the training group (*β* = −0.234, *p* = 0.042), which suggests that children with lower math skills prior to training show greater gains in growth mindset in response to training. These findings are consistent with greater improvements in WJ-III Math Fluency scores observed in children with math learning difficulties compared to typically developing children (see Supplementary Results for details). Together, our SEM reveals that cognitive training leads to better math performance in children who scored higher on growth mindset prior to training, providing evidence that growth mindset is associated with better math skills in response to cognitive training.

### Relation between training-induced changes in brain activation and growth mindset gains

**Fig. 2:**
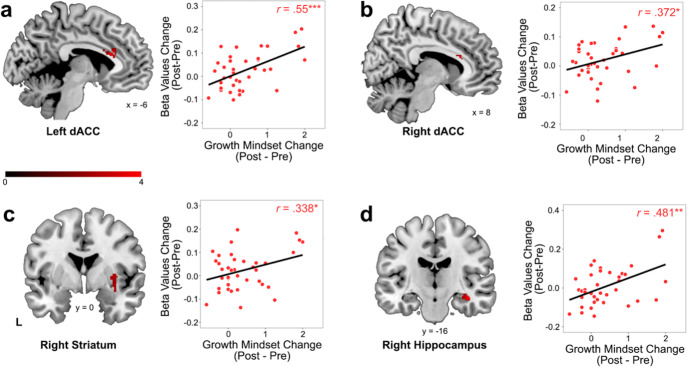
Plasticity of neural response associated with increases in growth mindset with cognitive training. Training-related increases in brain activation in the dorsal anterior cingulate cortex (dACC) on the (**a**) left and (**b**) right side, (**c**) right striatum (putamen), and (**d**) right hippocampus during more difficult math problem solving are significantly correlated with growth mindset gains in the training group. Color bar represents strength of association between changes in brain activation and growth mindset gains. Beta values of ROIs were extracted visualize the relationship between changes in growth mindset and changes in brain responses identified from whole brain analysis.

Our next goal was to investigate the neural correlates of training-induced changes in growth mindset associated with parametric modulation of math problem difficulty. Using an event-related parametric fMRI task design, our analysis assessed neurocognitive mechanisms of growth mindset gains associated with performance of more challenging math problems (see also *Methods*). We first examined changes in regional brain activation in relation to changes in growth mindset. We found that increases in brain activation in the dorsal ACC in both hemispheres, right dorsal striatum, and right hippocampus were correlated with increases in growth mindset in the training group (Fig. 2a–d; Table 1). Furthermore, gains in growth mindset remained significantly correlated with changes in brain responses in the right dorsal ACC and right hippocampus when controlling for age, IQ, and changes in math skills (Supplementary Table 7). Among the regions identified in the training group, no significant relation between changes in brain activation and changes in growth mindset was observed in in the control group (*p*s > 0.447). Interestingly, we did not observe changes in brain activation in the nucleus accumbens (ventral striatum) associated with gains in growth mindset in the training group (Supplementary Fig. 1). Together, these results uncover distinct neurobiological mechanisms underlying training-induced gains in growth mindset when solving more difficult math problems.

**Table 1:** Brain regions showing significant changes in neural response associated with gains in growth mindset.

	MNI Coordinates				
Region	*x*	*y*	*z*	Max *Z*	Cluster size
*Positive effect*					
dACC	−2	22	16	4.18	179
Left dACC^a^	−6	24	20	4.57	107
Right dACC^a^	12	26	22	3.04	44
Right hippocampus	38	−16	−16	3.53	147
Right striatum (putamen)	34	−2	−10	2.74	
*Negative effect*					
No significant clusters					

*dACC* dorsal anterior cingulate cortex.

^a^The left and right dACC subpeaks were identified using AAL masks.

### Relation between training-induced changes in functional connectivity and growth mindset gains

**Fig. 3:**
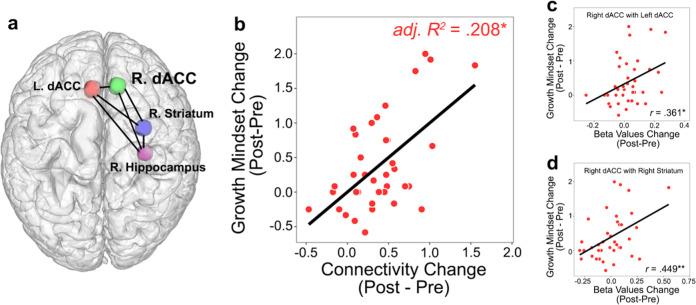
Plasticity of dorsal ACC–subcortical functional connectivity associated with increases in growth mindset with cognitive training. **a** Brain areas and relative strength of training-related changes in task-based connectivity associated with growth mindset gains. **b** Changes in connectivity amongst 4 brain regions (6 ROI-to-ROI links shown in (**a**)) jointly contributes to changes in growth mindset in the training group. **c**, **d** Changes in connectivity of (**c**) the right dorsal ACC with left dorsal ACC and those of (**d**) the right dorsal ACC with right striatum are significantly associated with changes in growth mindset in the training group.

We next examined whether changes in functional connectivity amongst four brain regions identified in regional activation analysis (bilateral dorsal ACC, right dorsal striatum, and right hippocampus; Fig. 3a) were related to changes in growth mindset with training. In a multiple regression model with changes in connectivity between nodes in a network of identified regions as predictors, we found that changes in functional connectivity amongst four brain regions jointly explained a significant amount variance in changes in growth mindset with training (Fig. 3b), *adj. R*^*2*^ = 0.208, *F*(6,31) = 2.615, *p* = 0.036. Such a relationship was not observed in the control group, *adj. R*^*2*^ = 0.047, *F*(6,10) = 1.131, *p* = 0.411. Additional analyses found that functional connectivity amongst the same set of brain regions (left and right dACC, right striatum and hippocampus) did not predict changes in math skills (measured by WJ-III Math Fluency) in the training group, *adj. R*^*2*^ = 0.105, *F*(6,31) = 1.726, *p* = 0.148. Furthermore, changes in functional connectivity of the nucleus accumbens with dACC, dorsal striatum, and hippocampus did not predict changes in growth mindset in the training group, *adj. R*^*2*^ = 0.069, *F*(4,33) = 1.682, *p* = 0.178.

We then examined the relation between changes in connectivity in each of the four regions of interest and changes in growth mindset scores, and found that increased connectivity of two individual links of the right dorsal ACC were positively correlated with changes in growth mindset with training: right dorsal ACC - left dorsal ACC, *r*(36) = 0.361, *p* = 0.026 (0.078, FDR-adjusted), and right dorsal ACC - right dorsal striatum, *r*(36) = 0.449, *p* = 0.005 (0.028, FDR-adjusted) (Fig. 3c, d; Supplementary Table 8). These associations were not significant in the control group (Supplementary Table 8). Taken together, these results further elucidate that plasticity of dorsal ACC and striatal response and inter-connectivity supports children’s growth mindset gains in response to cognitive training.

## Discussion

We examined whether cognitive training designed to promote academic learning in early elementary school children could in parallel enhance growth mindset, and investigated brain mechanisms underlying individual differences in growth mindset gains with training. Our analysis revealed three major findings. First, we found that cognitive training led to improvements in growth mindset with greater benefits among those who had lower levels of growth mindset prior to training. Second, children with higher levels of growth mindset prior to training showed better math performance in response to training. Third, growth mindset gains were associated with plasticity of neural response and connectivity involving dorsal ACC, striatum, and hippocampus regions that support cognitive control, motivation, and memory. Our findings demonstrate that growth mindset can be enhanced by cognitive training and, furthermore, provide new insights into the neurobiological mechanisms underlying malleability of growth mindset.

Our study provides direct evidence that a tutoring-based training program targeting foundational cognitive skills in children leads to increases in growth mindset—a belief that one’s intelligence can change with effort that is associated with increased desire to learn, positive views of effort, and willingness to take on challenges^5^. Our training program, which was individually tailored to students’ learning needs with the goal of mastering fundamental number knowledge, effectively improved both numerical skills^29^ and growth mindset in children. Similar to our behavioral findings, a previous study with older adults also found increased growth mindset following cognitive intervention, which enhanced learning in parallel^14^. Our findings suggest that a positive learning experience focused on mastery of learning materials may facilitate children to endorse growth mindset by fostering beliefs that their abilities can be improved through effort and reducing attribution of their success or failure to fixed traits^5,30^. These findings are also consistent with a meta-analysis pointing to a link between mastery-oriented learning goals and growth mindset^31^, suggesting that a supportive learning environment can lead to simultaneous improvements in academic performance and growth mindset.

We found a stronger negative correlation between gains in growth mindset and growth mindset prior to training in the training group, compared to the control group. This finding points to greater benefits from the training program for children with lower levels of growth mindset which is commonly associated with lower academic achievement. Cross-lagged panel analysis revealed that children with lower math skills prior to training had higher endorsement of growth mindset after training. Furthermore, cognitive training led to improvements in a wider range of math skills, extending beyond quantity discrimination ability^29^ to arithmetic problem solving skills, particularly in children with math learning difficulties. Thus, it is possible that our cognitive training program provided additional opportunities for low-achieving children to endorse more growth mindset through enhanced learning in core number knowledge^32,33^. Convergent with our findings, previous growth mindset interventions have also observed greater benefits in students with lower achievement^7,34^. Together, these findings suggest that cognitive training can enhance both academic performance and growth mindset.

Structural equation modeling of longitudinal behavioral data revealed that growth mindset prior to training predicted higher math skills with training, even after controlling for math skills prior to training. Importantly, the stronger positive impact of growth mindset on math skills observed in the training, compared to control, group indicates that the influence of growth mindset on achievement is strengthened by cognitive training. These findings converge on related literature pointing to a positive correlation between growth mindset and performance gains in response to training in other domains such as working memory and cognitive control^14–16^. Our findings suggest that a training program that focused on mastery of contents can positively influence learning in children. Likewise, interventions focused on enhancing growth mindset are thought to have positive impact on academic achievement^5,7,35^. These findings are broadly consistent with the notion that motivational aspects of learning are critical for individuals’ success^36^. Critically, longitudinal assessments of both growth mindset and cognitive abilities have been surprisingly rare. Our unique longitudinal training study combined with structural equation modeling overcame limitations of cross-sectional studies and demonstrated the links between growth mindset and cognitive skill acquisition in a more rigorous manner. Our findings also provide new insights into whether growth mindset is associated with individual differences in response to interventions in an academically relevant domain.

Our final goal was to explore potential brain mechanisms underlying growth mindset gains in response to cognitive training in children. Here we found that growth mindset gains were associated with increased neural response and connectivity of the dorsal ACC, striatum, and hippocampus. Notably, dorsal ACC connectivity with striatum emerged as the strongest individual circuit predicting growth mindset gains, which suggests a central role for plasticity of cortico-striatal circuity in driving growth mindset changes. The dorsal ACC is important for cognitive control processes, involving volitional action and implementation and correction of action plans^37–39^, which are instrumental in regulating attention and enabling learners to stay task-focused in the presence of obstacles^40,41^. The engagement of these processes is consistent with the notion that growth mindset allows individuals to persist in response to setbacks^5^. Behavioral studies have suggested that growth mindset is associated with self-regulated learning, including persistence, planning, and monitoring^3,31^. Studies using event-related potentials have also linked growth mindset with neural response to cognitive control and error-monitoring processes that facilitate “learning from mistakes”^22^. Our findings suggest that greater engagement of dorsal ACC together with striatum, implicated in value-based action selection^42,43^, may facilitate more effective goal-directed actions to support learning and growth mindset.

Theories of mindset have posited distinct motivational and volitional phases involving goal selection and implementation^44^. Importantly, dorsal ACC circuits play a central role in both these processes and are crucial for regulating cognitive control which is effortful and intrinsically costly^45^. Dorsal ACC and cortico-striatal circuits are also central to allocation of cognitive resources based on evaluation of the expected value of control, which regulates how much control to exert by weighing effort costs against potential rewards or performance gains^46^. Consistent with this view, a recent behavioral study found that cognitive control skills were malleable and could be improved through training^47^. Taken together, these findings suggest that children who endorse more growth mindset in response to training are more likely to engage in action selection toward learning goals, which in turn facilitates cognitive skill acquisition.

Although the behavioral component of our study was adequately powered, one limitation is the smaller neuroimaging sample size of the control group. Consequently, our analysis of the relation between training-induced changes in brain activation and growth mindset gains was limited to the training group. Further studies with larger neuroimaging samples in the control group are needed to determine the specificity of our findings. Another potential limitation is that we were not able to identify factors that contributed to changes in growth mindset in the control group. Follow-up studies are needed to clarify whether positive learning experiences in regular classrooms may also contribute to gains in growth mindset. Future studies will also benefit from appropriate active control groups that are closely matched to training group in terms of participants’ expectation of improvements^48^. These enhancements may also lead to more precise characterization of the neurocognitive mechanisms underlying growth mindset gains in children with varying levels of cognitive abilities.

In conclusion, our study demonstrates that a cognitive training program designed to strengthen academically-relevant foundational skills can also enhance growth mindset in 7–10-year-old children. We suggest that plasticity of cortico-striatal circuits involved in volitional cognitive control and action selection is a key neurobiological mechanism underlying cognitive-training-induced changes in growth mindset. Findings provide support for the positive role of growth mindset in academic learning and achievement. We suggest that interventions that combine growth mindset and cognitive training may be especially beneficial for students with learning difficulties. More generally, our findings may inform evidence-based growth mindset interventions based on notions of brain plasticity^5,7,49^.

## Methods

### Participants

Ninety-six participants were initially recruited in the Great San Francisco Bay area to participate in our training study. Participants were right-handed and had no history of psychiatric illness or neurological disorders. All protocols were approved by the Stanford University Institutional Review Board and were performed in accordance with the American Psychological Association Code of Conduct. Written informed consent was obtained from the parents of the children.

The current study focused on growth mindset changes and their neural basis in children and therefore, we only included individuals who had complete behavioral and brain imaging at both pre- and post-visits, resulting in a total of 79 children (45 females; age range = 6.76–10.02 years old, *M* = 8.20, SD = 0.65 at pre-visit). Among them, 52 children participated in training and 27 children were part of a no-contact control group, which controlled for business-as-usual schooling experience^50^. Children in the no-contact control group completed all components of the study except the 4-week training (see also study design in Fig. 1a). The two groups did not differ in age, gender, IQ, growth mindset, or math problem solving tasks prior to training (Supplementary Table 1).

Because no previous studies have directly examined growth mindset gains in response to cognitive training in children, population-based estimate of effect size^51^ could not be used for power analysis. Based on previous tutoring-based training studies with children (range of Cohen’s *d*: 1.1–1.3)^52,53^, we estimated that a sample size of >26 would achieve a power of >99% for analysis of gains in growth mindset. Observed effect sizes and power from the current study are reported in Supplementary Results.

For investigations of brain plasticity underlying changes in growth mindset in response to cognitive training, we used data from 38 participants in the training group with high-quality fMRI and behavioral data at both pre- and post-visits. A total of 14 (out of 52) participants were excluded for the following reasons: (i) 12 participants did not have high-quality fMRI data at pre- and/or post-visits; (ii) 1 participant was excluded due to low task accuracy (<40%) in the fMRI task at both visits; and (iii) 1 participant was excluded due to invalid or incomplete neuropsychological assessments at post-visit. The sample size in the training group (*n* = 38) was determined adequate (estimated power > 83%) for brain-behavior analysis based on previous fMRI studies of tutoring-based intervention in children (range of Cohen’s *d*: 1.0–1.2)^54,55^ (for more details on observed effect sizes and power, see also Supplementary Results). After applying the same exclusion criteria, a total of 17 participants with high-quality fMRI data were included in the control group. The two groups did not differ in age, gender, IQ, growth mindset, or arithmetic tasks at pre-visit (*p*s > 0.05). Due to the modest sample size in the control group, our fMRI data analysis in this group was limited to characterizing the specificity of brain-behavior relations observed in the training group.

#### Subgroups of subjects based on math ability

The training group included children with a wide range of math abilities, based on their scores on Math Fluency subtest of Woodcock-Johnson III Tests of Early Cognitive and Academic Development (WJ-III)^56^ assessed prior to training. A total of 20 children scoring lower than 90 (below 25th percentile) were identified as having math learning difficulties (MLD) and the rest of the 32 children scoring 90 or higher were identified as typically developing (TD) children.

### Behavioral measures

#### Neuropsychological assessment

A comprehensive standardized battery of neuropsychological assessments was administered to each participant, including a demographic questionnaire, the Wechsler Abbreviated Scale of Intelligence (WASI)^57^ to assess IQ, and the WJ-III^56^ to determine mathematical abilities. In addition to Math Fluency, we administered Calculation, Applied Problems, Letter-Word Identification, and Word Attack subtests from the WJ-III, which were not analyzed in the current study. Two alternative versions of the WJ-III Math Fluency subtest were administrated at pre- and post-visits to minimize test-retest practice effects in assessment of children’s improvements in math skills.

#### Growth mindset survey

Children’s growth mindset was assessed by a brief 12-item survey, adapted from theories of intelligence scale^58^ at both pre- and post-visits. The items included questions about participants’ growth mindset in math, reading, and intelligence in general (Supplementary Table 2). Participants were asked to respond on a 6-point Likert scale of “very very disagree,” “disagree,” “somewhat disagree,” “somewhat agree,” “agree,” and “very very agree.” Half of the items were negatively phrased and were reverse coded. The mean score of these 12 items indexed the level of growth mindset, ranging from 0 to 5 with 5 indicating the highest level of growth mindset. The internal reliability of this scale in our sample at pre-test, Cronbach’s alpha = 0.82, was comparable to that of original theories of intelligence scale, 0.78^6^.

### Training protocol

To improve fundamental understanding of quantity in children, we used a one-on-one tutoring-based training program (3 days/week, for ~60 min/day) designed for early elementary school children (Fig. 1a). From weeks 1 to 4, children in the training group progressively acquired fundamental number knowledge. Tutoring activities were carefully chosen to improve cognitive abilities associated with counting, comparing, and ordering numbers in non-symbolic (arrays of dots) and symbolic (Arabic numerals) formats. To promote mastery-oriented learning which is closely aligned with growth mindset, as opposed to performance-oriented approaches^5,59^, children were encouraged to learn and received positive feedback upon completion of activities, rather than being evaluated for their performance levels. More details of the tutoring procedures in the training program and children’s gains in number knowledge in response to training can be found in a recent publication^29^.

### fMRI task

During both pre- and post-visits, children completed two runs of math problem-solving (addition) task (Fig. 1a) in the MRI scanner. Given previously shown associations between growth mindset or positive attitude and math achievement^6,7,28^, we chose to examine the neurobiological correlates of growth mindset during math problem solving. During each trial of our math problem-solving task, an addition problem was presented for 6 s, followed by a proposed solution which was presented for 1 s. Participants had a time window of up to 10.8 s (starting from the presentation of the solution) to indicate whether or not the proposed solution is correct. A total of 24 single-digit problems with operands from 2 to 9 (excluding ties) were presented in each run with the order of problems randomized across participants. Half of proposed solutions were valid, whereas the other half were invalid with solutions deviating from the correct answer by ±1 or ±2 units. The difficulty of each addition problem (“problem difficulty”) was estimated by the correct answer to the problem, ranging from 5 to 17.

### Behavioral data analysis

#### Effects of cognitive training on growth mindset

To address our first research question about whether children’s growth mindset scores change in response to cognitive training, we conducted a mixed ANCOVA with group (training, control) as a between-subject factor, time (pre-, post-training) as a within-subject factor, and pre-training (i.e., baseline) growth mindset score as a covariate of no interest to control for pre-existing differences in growth mindset. Additional two-tailed paired sample *t*-tests across pre- and post-visits in the training (or control) group examined whether and how 4 weeks of training (or business-as-usual classroom activities) lead to changes in growth mindset scores. Two-tailed two-sample *t*-tests at both pre- and post-visits determined whether the two groups of children (training vs. control) demonstrate similar or different levels of growth mindset at each time point. To further address whether children’s pre-training levels of growth mindset influence the degree of changes in growth mindset through cognitive training, we examined how changes in growth mindset are associated with individual differences in children’s initial levels of growth mindset, using Pearson’s correlation. Fisher’s *Z* test was used to test difference in correlation coefficients between training and control groups to examine whether the group moderates the relationship between growth mindset at pre-visit and changes in growth mindset. All the analyses, including estimates of effect sizes (Cohen’s *d*, *η*^2^, *r*), were conducted in R (version 3.6.1; Team^60^).

#### Effects of growth mindset on academic achievement

In order to assess the effect of growth mindset on academic achievement through cognitive training, we used structural equation modeling (SEM) to conduct a cross-lagged panel analysis (Fig. 1d, e). A longitudinal design provided a unique opportunity to examine the relation between growth mindset on cognitive abilities over time. WJ-III Math Fluency scores were used as the measure of math skills in children, which has been shown to be associated with academic achievement^56^. In this model, we assumed auto-correlational effects for both growth mindset and math skills (e.g., the effect of growth mindset at pre-visit on growth mindset at post-visit), cross-sectional relationships between growth mindset and math skills at each visit (e.g., the association between growth mindset and math skills at pre-visit), and most importantly, cross-lagged effects between growth mindset and arithmetic skills across visits (e.g., the effect of growth mindset at pre-visit on arithmetic skills at post-visit). Cross-lagged effects in this model were independent from auto-correlational and cross-sectional effects, thereby providing more accurate estimates of unique influence of growth mindset on academic achievement over time through training. We first examined this cross-lagged effect of growth mindset at pre-visit on math skills at post-visit in each group (training, control) to determine which group shows a significant cross-lagged effect. Next, we conducted a model-fit comparison in a multi-group analysis by comparing a baseline (unconstrained) model in which all parameters were free to estimate with a constrained model in which the cross-lagged effect from growth mindset at pre-visit on math skills at post-visit was set to be equal in both training and control groups. A significant difference between the model fits of baseline and constrained models would indicate that the influence of growth mindset at pre-test on math skills on post-visit is different between the two groups. SEM analyses were conducted with the *Lavaan* package (version 0.6-5; Rosseel^61^) in R (version 3.6.1; Team^60^).

### fMRI data analysis

#### MRI acquisition and preprocessing

Functional brain images were acquired on a 3 T Signa scanner (General Electric, Milwaukee, WI) using a custom-built head coil at Stanford University Lucas Center for Imaging. Head movement was minimized during the scan by cushions placed around the participant’s head. A total of 31 axial slices (4.0 mm thickness, 0.5 mm skip) parallel to the anterior commissure-posterior commissure line and covering the whole brain were imaged using a 2D gradient echo spiral in-out pulse sequence^62^ with the following parameters: repetition time = 2000 ms, echo time = 30 ms, flip angle = 80°, 1 interleave. The field of view was 220 mm and the matrix size was 64 × 64, providing an in-plane spatial resolution of 3.4375 mm. To reduce blurring and signal loss from field inhomogeneity, we used an automated high-order shimming method based on spiral acquisitions before acquiring fMRI scans^63^.

Functional MRI data were analyzed using SPM12^64^. The first 5 volumes were not analyzed to allow for T1 equilibration. A linear shim correction was applied separately for each slice during reconstruction^65^. Images were realigned to correct for movement and slice acquisition timing. Images were then spatially normalized to Montreal Neurological Institute (MNI) space and smoothed with a 6 mm full-width half maximum Gaussian kernel to decrease spatial noise prior to statistical analysis. Translational movement in millimeters (*x*, *y*, *z*), and rotational motion in degrees (pitch, roll, yaw) were calculated based on the SPM12 parameters for motion correction of the functional images of each run of the addition task. We excluded any run with movement greater than 10 mm in any of the *x*, *y*, *z* directions, pitch, yaw, roll rotations, or mean scan-to-scan displacement of movement exceeding 0.5 mm. Movement-related deviant volumes (movement >0.5 voxels or spikes in the global signal >5%) were interpolated with two adjacent scans by a de-spiking procedure similar to those implemented in AFNI^66^. No >15% of total volumes per run were interpolated in either visit of any participant.

#### Individual statistics

Since no control condition was included in the addition task, task-related brain responses were estimated with a parametric approach in the framework of general linear model implemented in SPM12^64^. An event-related parametric fMRI task design allowed us to examine the effects of task difficulty, controlling for potential confounds such as visual perception and motor response. For each run at the individual level, problem difficulty of all addition problems was coded as a modulating parameter to examine the positive relationship between brain response and problem difficulty. Here, higher levels of brain responses indicated greater engagement during completion of more difficult math problems. Movement parameters of each run estimated from the preprocessing stage were included as covariates of no interest. The onset of each trial was convolved with a canonical hemodynamic response function and a temporal derivative to account for voxel-wise latency differences in hemodynamic response. Voxel-wise estimates associated with the parameter of problem difficulty in the correct trials during the problem presentation were generated for each run of each participant at both pre- and post-visits. For each visit (pre or post) in each participant, one or two runs of available high-quality addition task data were included in further analysis. High-quality addition task data met criteria for movement (see MRI acquisition and preprocessing) and performance on the addition task (>40% accuracy per run) to ensure attention to task and sufficient number of trials for data analysis.

#### Group-level statistics

At the group level, we examined changes in brain activation (post-visit – pre-visit) associated with changes in growth mindset scores across individuals in the training group, using an *F*-test with contrast images of parametric estimates based on problem difficulty from individual statistics. In this *F*-test, we modeled time points (pre-visit, post-visit) as a within-subject factor and the difference score of growth mindset (post-visit – pre-visit) as a covariate. We examined both positive and negative contrasts of the interaction effects between time points and the difference score of growth mindset. The whole-brain analysis identified clusters of activation using a height threshold of *p* < 0.01, with family-wise error (FWE) corrections for multiple comparisons at the cluster level (*p* < 0.01; spatial extent of 128 voxels based on Monte Carlo simulations).

#### ROI selection and analysis

To visualize changes in brain activation associated with changes in growth mindset and ensure results from whole brain analysis are not driven by outliers, we created functionally defined regions of interest (ROIs) with a diameter of 6 mm centering on the peak coordinates of brain regions showing significant results from regional activation analysis, which included dorsal ACC, right striatum (putamen), and right hippocampus (Table 1). For the cluster of dorsal ACC that crossed the two hemispheres, we used anatomical masks of the left and right cingulate gyri from the Automated Anatomical Labeling (AAL) atlas^67^ to identify local peaks in the left and right hemispheres separately. In addition, to examine the potential role of ventral striatum, which was not identified in our regional activation analysis, in supporting growth mindset, we used parcellations of nucleus accumbens (NAc) from Brainnetome^68^ as a priori ROI. Pearson’s correlation was used to examine the strength of association between changes in brain activation in ROIs and changes in growth mindset. Additional ROI-based analyses were conducted in the control group with the ROIs defined from the training group to examine whether the observed associations are present without training. Finally, change in beta values from pre- to post-training in these ROIs was used to further clarify whether changes in brain activation are correlated with changes in growth mindset even when controlling for changes in age, IQ (WASI Full Scale IQ), and math skills (WJ-III Math Fluency).

#### Multivariate network analysis

To further examine plasticity of connectivity amongst key regions associated with changes in growth mindset during math performance, we first used the general psychophysiological interaction (gPPI) method^69^ and examined task-dependent functional connectivity associated with the parameter of problem difficulty. Four ROIs were selected from our regional activation analysis: the left and right dorsal ACC, right striatum (putamen), and right hippocampus. For each participant at each visit, a model was computed with the parameter of problem difficulty, each ROI time course, and their interaction term with only correct trials included. The beta values of the interaction term were then extracted for all pairs of 4 ROIs (6 ROI-to-ROI links; Fig. 3a) for each visit of each participant. In a multivariate network regression analysis, we first examined whether changes in task-dependent functional connectivity (post-visit – pre-visit) between all 4 ROIs could predict changes in growth mindset scores (post-visit – pre-visit). Next, we examined associations between changes in each individual ROI-to-ROI link and changes in growth mindset scores. Pearson’s correlation was used to examine associations between changes in functional connectivity for each ROI-ROI link and changes in growth mindset. Analyses were performed for both training and control groups to address training-specific brain plasticity associated with growth mindset gains. Finally, to assess the potential role of NAc in the ventral striatum in growth mindset, we additionally examined whether its functional connectivity with the 4 ROIs could jointly predict training-induced changes in growth mindset.

### Observed effect sizes and power

Based on observed effect sizes of various analyses in the training group, we estimated observed power in the training group at the alpha level of 0.05. For the control group, we used effect sizes from the training group to estimate whether the available sample size in the control group would have enough power to detect the same effect sizes at an alpha level of 0.05. All power analyses were conducted in R (version 3.6.1; Team^60^ using the package “pwr” (version 1.3-0)^70^ and “pwrSEM” (version 0.1.2)^71^.

### Reporting summary

Further information on research design is available in the Nature Research Reporting Summary linked to this article.

## Supplementary information

Supporting Information

Reporting Summary

## Data Availability

Data used to support the findings of the current study are not openly available to ensure confidentiality of data acquired from participants and are available from corresponding authors upon reasonable request for research purposes within the agreement of the informed consent approved by the Institutional Review Board.

## Code availability

Data were analyzed using R 3.6.1, SPM12, and Matlab 9.2-9.8. Data analysis scripts used for the current study are available on GitHub (https://github.com/scsnl/Chen_npjSoL_2022).
