# Supplementary material for: Cognitive training enhances growth mindset in children through plasticity of cortico-striatal circuits
Source: NPJ Sci Learn. 2022 Nov 12;7:30. doi: 10.1038/s41539-022-00146-7 (PMC9653476; doi:10.1038/s41539-022-00146-7)
Supplement: Supplementary file 1 — Supporting Information [file 41539_2022_146_MOESM1_ESM.pdf]

Supplementary Information

Cognitive training enhances growth mindset in children through plasticity of cortico-striatal circuits

Supplementary Results …………………………………………………………….…….*p.* 2

Supplementary Figure 1 ……...……………………………………………….….….….…..…*p.* 6

Supplementary Tables 1 to 10 ..……………………………………………….….….….… *p.* 7

Supplementary References ………………………………………………………….…. *p.* 17

Supplementary Results

Observed effect sizes and power: behavior data analyses

We observed that the training group with a sample size of 52 in the behavioral data analyses (*post-pre change in growth mindset ["growth mindset gain"]*, *relation between growth mindset gain and growth mindset prior to training*, and *structural equation modeling [SEM] of the link between growth mindset prior to training and math achievement post-training*) was estimated to have a power ranging from .62 to >.99. The control group with a sample size of 27 (24 for SEM analysis) in behavioral data analyses was estimated to have a power ranging from .44 to >.99. Observed effect sizes and power can be found in **Supplementary Table 3**.

Observed effect sizes and power: brain-behavior relations

The training group with a sample size of 38 in neuroimaging data analyses (*relation between post-pre change in brain activation or connectivity and growth mindset gain*) was estimated to have a power ranging from .56 to .96. The control group with a sample size of 17 was estimated to have low-to-medium power for brain-behavioral analysis, ranging from .27 to .66. Observed effect sizes and power can be found in **Supplementary Table 3**.

Changes in children's quantity discrimination ability following four weeks of cognitive training

Our cognitive training program focused on strengthening children's fundamental number knowledge. Children's training gains were assessed by performance on symbolic quantity discrimination task administered before and after training (see Chang et al. (2022) for details of task design). One participant did not have symbolic quantity discrimination task data. A total of

78 participants (52 children in the training group, 26 children in the control group) were included in analysis. A 2-way ANOVA with time (pre-, post-training) and group (training, control) revealed a significant main effect of time ($F(1,76) = 23.810$, $p < .001$) and a significant interaction between time and group on symbolic quantity discrimination task performance ($F(1,76) = 12.160$, $p < .001$). No significant main effect of group was observed ($F(1,76) = 1.301$, $p = .258$). Post-hoc analysis showed that performance gains were significant in the training group ($t(51) = -5.998$, $p < .001$) but not in the control group ($t(25) = .031$, $p = .976$).

We next examined whether training gains were similar or different between children with MLD ($N = 20$; see **Methods**) and TD children ($N = 32$) in the training group. In a 2-way ANOVA, we found a significant main effect of time ($F(1,50) = 35.701$, $p < .001$) and no significant main effect of group (MLD, TD) or interaction between time and group (Fs < .988; ps > .324). Significant training-induced improvements were observed in both groups (MLD: $t(19) = -2.802$, $p = .011$; TD: $t(31) = -5.559$, $p < .001$). These findings indicate that training induced changes in quantity discrimination ability across children with and without math learning difficulties, consistent with our recent publication (Chang et al., 2022).

Changes in children's addition problem solving skills following four weeks of cognitive training

We further examined whether children's performance on an event-related fMRI task involving addition problem solving changes in response to training. Eleven participants did not have valid addition fMRI task data. A total of 68 participants (45 children in the training group, 23 children in the control group) were included in analysis. A 2-way ANOVA with time (pre-training, post-

training) and group (training, control) on addition task accuracy revealed a marginally significant main effect of time ($F(1,66) = 3.553$, $p = .064$) and a significant interaction between time and group ($F(1,66) = 4.373$, $p = .040$). No significant main effect of group was observed ($F(1,66) = .992$, $p = .323$). Post hoc analysis showed that improvements were significant in the training group ($t(44) = -2.558$, $p = .014$) but not in the control group ($t(22) = -.728$, $p = .474$).

Additional 2-way ANOVA comparing children with MLD ($N = 16$) and TD children ($N = 29$) in the training group revealed a marginally significant interaction between time and group (MLD, TD), $F(1,43) = 3.313$, $p = .076$ (**Supplementary Table 9**). Significant training-induced improvements were observed in the MLD subgroup ($p = .013$). These findings suggest that our cognitive training protocol was effective in enhancing addition problem solving skills beyond quantity discrimination ability that children were trained on.

Changes in children's WJ-III Math Fluency scores following four weeks of cognitive training

Finally, in addition to quantity discrimination and addition problem solving skills, we examined whether our training protocol improved children's math skills more broadly on the Math Fluency subtest of the WJ-III (a standardized assessment of arithmetic fluency). Five participants did not have valid WJ-III Math Fluency subtest score. A total of 74 participants (51 children in the training group, 23 children in the control group) were included in analysis. A 2-way ANOVA with time (pre-, post-training) and group (training, control) on WJ-III Math Fluency standardized scores showed a marginally significant main effect of time ($F(1,72) = 3.273$, $p = .075$) and a significant main effect of group ($F(1,72) = 4.682$, $p = .034$). No significant interaction between time and group was observed ($F(1,72) = .816$, $p = .369$).

We additionally assessed training-related changes in WJ-III Math Fluency scores in children with MLD (*N* = 20) and TD children (*N* = 31). Here, a 2-way ANOVA showed a significant interaction between time and group (MLD, TD), $F(1,49) = 7.268$, $p = .010$ (**Supplementary Table 10**) with significant training-induced improvements observed in the MLD subgroup ($p = .005$). These findings suggest that training was particularly effective in children with math learning difficulties who may have benefited from scaffolding knowledge of numbers to arithmetic problem solving.

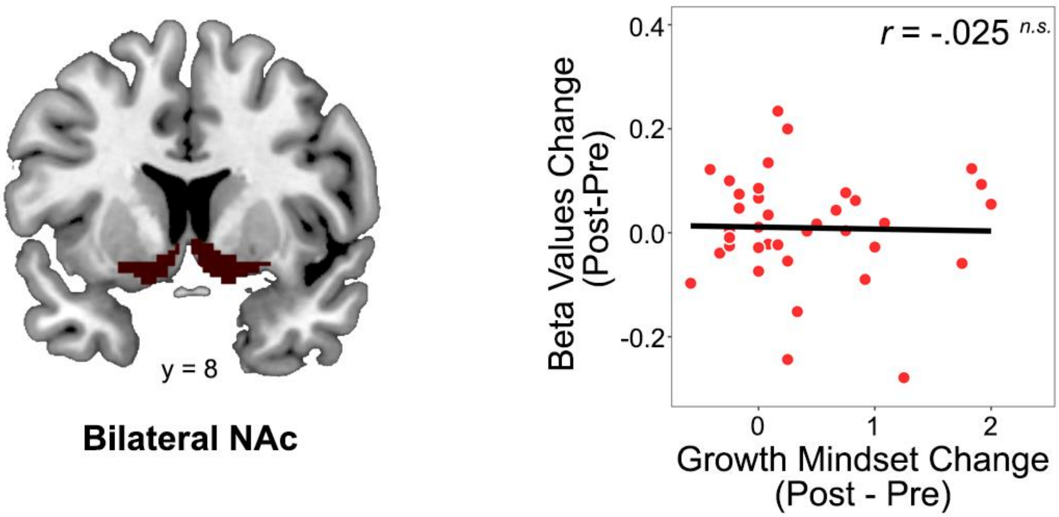

Supplementary Figure 1. Change in growth mindset was not significantly associated with change in brain activity in the bilateral nucleus accumbens (NAc) in the training group. *n.s.* = not significant.

Supplementary Table 1. Demographic and behavioral profiles of training and control groups

	Training (n = 52)	Control (n = 27)	Chi-square test or two-sample t-test		
Gender (Female : Male)	29 : 23	16 : 11	χ^2 = .003		p = .954
	Mean (SD)	Mean (SD)	t	df	p
**Time 1 (Pre)**					
Age	8.18 (.65)	8.26 (.64)	-.525	77	.601
FSIQ	107.65 (12.80)	109.11 (11.02)	-.503	77	.617
Growth mindset	4.07 (.73)	3.94 (.83)	.695	77	.489
Math fluency	96.10 (12.97)	100.70 (10.29)	-1.601	77	.113
Addition accuracy	.80 (.17)	.86 (.11)	-1.622	66	.109
**Time 2 (Post)**					
Age	8.36 (.66)	8.49 (.67)	-.828	77	.410
Growth mindset	4.52 (.49)	4.21 (.79)	2.142	77	**.035**
Math fluency	97.39 (12.91)	104.91 (13.77)	-2.272	72	**.026**
Addition accuracy	.85 (.12)	.85 (.10)	.116	66	.908

Supplementary Table 2. Adapted theory of intelligence scale (Dweck, 1999) to assess growth mindset in children

	Very Very Disagree	Disagree	Somewhat disagree	Somewhat agree	Agree	Very Very Agree
You can change how good you are at reading.	0	1	2	3	4	5
If you work really hard you can change how good you are at math.	0	1	2	3	4	5
If you work really hard you can get smarter.	0	1	2	3	4	5
You can change how good you are at math.	0	1	2	3	4	5
You can't change how smart you are.	0	1	2	3	4	5
If you work really hard you can change how good you are at reading.	0	1	2	3	4	5
You can't change how good you are at reading.	0	1	2	3	4	5
Even if you work really hard you can't get smarter.	0	1	2	3	4	5
Even if you work really hard you can't change how good you are at math.	0	1	2	3	4	5
You can change how smart you are.	0	1	2	3	4	5
You can't change how good you are at math.	0	1	2	3	4	5
Even if you work really hard you can't change how good you are at reading.	0	1	2	3	4	5

Notes: This questionnaire assessed children’s growth mindset in general intelligence as well as specific academic domains, reading and math. Items 1-4, 6, and 10 were statements about growth mindset and items 5, 7-9, 11, and 12 were statements about fixed mindset. To obtain a total score for growth mindset, fixed mindset items were reverse-coded.

Supplementary Table 3. Observed effect sizes and power in training and control groups across various analyses

Analysis	Statistical Test	Training Group (Cohen's *d*/power)	Control group (Cohen's *d*/power)
**Behavioral**		(*n* = 52)	(*n* = 27; 24[1])
Post-Pre change in growth mindset	Paired-sample *t* test	.698/.998	.331/.937
Growth mindset gain correlates with mindset at pre-visit	Correlation (*r*)	2.280/.999	.736/.998
SEM (mindset at pre-visit → math skills at post-visit)	Path coefficient	.442/.620	.448/.440[1]
**Neuroimaging**		(*n* = 38)	(*n* = 17)
Activation in the left dACC	Correlation (*r*)	1.317/.959	.387/.659
Activation in the right dACC	Correlation (*r*)	.802/.647	.120/.322
Activation in the right striatum	Correlation (*r*)	.718/.558	.402/.271
Activation in the right hippocampus	Correlation (*r*)	1.097/.880	.402/.520
Connectivity between the right dACC and left dACC	Correlation (*r*)	.774/.619	.173/.305
Connectivity between the right dACC and right striatum	Correlation (*r*)	1.005/.824	.530/.458

[1]Data available from 24 children were included in the control group for SEM analysis.

Supplementary Table 4. Correlation matrix in the training group

	1	2	3	4	5	6	7	8	9
**Time 1 (Pre)**									
1 Age	-								
2 FSIQ	-.039	-							
3 Growth mindset	.026	.303*	-						
4 Math fluency	-.125	.155	.072	-					
5 Addition accuracy	.167	.477***	.27#	.449**	-				
**Time 2 (Post)**									
6 Age	.988***	-.022	.052	-.15	.156	-			
7 Growth mindset	-.272#	-.058	.504***	-.197	-.059	-.247#	-		
8 Math fluency	-.028	.296*	.265#	.693***	.595***	-.058	-.092	-	
9 Addition accuracy	.265#	.294#	.247	.218	.628***	.254#	.15	.384*	-
10 Growth mindset gains	-.237#	-.388**	-.752***	-.232#	-.357*	-.248#	.19	-.372**	-.165

Notes: # $p < .10$; * $p < .05$; ** $p < .01$; *** $p < .001$.

Supplementary Table 5. Correlation matrix in the control group

	1	2	3	4	5	6	7	8	9
**Time 1 (Pre)**									
1 Age	-								
2 FSIQ	.144	-							
3 Growth mindset	-.047	.111	-						
4 Math fluency	.08	.325#	-.232	-					
5 Addition accuracy	-.182	.204	.249	-.259	-				
**Time 2 (Post)**									
6 Age	.993***	.158	-.018	.069	-.166	-			
7 Growth mindset	.064	.198	.863***	-.08	.132	.081	-		
8 Math fluency	.066	.379#	-.426*	.728***	-.09	.054	-.263	-	
9 Addition accuracy	.033	.32	-.052	.176	.47*	.028	.06	.167	-
10 Growth mindset gains	.211	.15	-.345#	.303	-.229	.186	.175	.332	.187

Notes: # $p < .10$; * $p < .05$; ** $p < .01$; *** $p < .001$.

Supplementary Table 6. Model comparison between baseline and constrained models in multi-group analysis

	df	AIC	BIC	Chi-square	Chi-square difference	*df* difference	*p*
Baseline model	0	1456.4	1522.7	.000			
Constrained model	1	1458.8	1522.8	4.433	4.433	1	.035

Abbreviations: AIC = Akaike information criterion; BIC = Bayesian information criterion.

Supplementary Table 7. Relationship between growth mindset gain (post-pre) and changes in brain activation (post-pre) when controlling for effects of age, IQ and changes in math skills (post-pre)

	Right dACC	Left dACC	Right striatum	Right hippocampus	Bilateral NAc
**Training group**					
Age at pre	.006	-.020	.017	.042	.018
IQ	-.001	-.001	.000	.001	.002
Math skill gains (Post – Pre)	.001	.000	-.001	-.001	-.001
Growth mindset gains (Post – Pre)	**.062****	.023	.044#	**.084****	.011
**Control group**					
Age at pre	.006	.005	.032	.029	.012
IQ	.003	.001	.000	**.004***	-.002
Math skill gains (Post – Pre)	.001	.000	-.002	-.002	.002
Growth mindset gains (Post – Pre)	.033	.013	-.033	.044	**-.131****

Notes: Values represent beta values for each predictor in multiple regression analysis. IQ was assessed by Full Scale IQ from WASI. Math skill was measured by Math Fluency from WJ-III.

# $p < .10$, * $p < .05$, ** $p < .01$.

Supplementary Table 8. Bivariate correlation between changes in connectivity for each ROI-to-ROI link and growth mindset gains

Link	Training group			Control group		
	r	*p* (unc.)	*p* (FDR corr.)	*r*	*p* (unc.)	*p* (FDR corr.)
R.dACC-L.dACC	.361	**.026**	***.078***	.086	.743	.939
R.dACC-R.Striatum	.449	**.005**	**.028**	.256	.321	.642
R.dACC-R.Hippocampus	.160	.339	.508	.462	***.062***	.370
L.dACC-R.Striatum	.303	***.065***	.130	.027	.917	.939
L.dACC-R.Hippocampus	.012	.944	.944	.256	.321	.642
R.Striatum-R.Hippocampus	-.066	.693	.832	-.020	.939	.939

Abbreviations: dACC = dorsal anterior cingulate cortex; L = left; R = right; unc. = uncorrected; FDR corr. = FDR corrected.

Supplementary Table 9. ANOVA and paired *t*-tests for changes in addition fMRI task accuracy in MLD and TD groups in the training group

	F	df_1, df_2	*p*
Omnibus ANOVA			
Group (MLD vs. TD)	5.118	1, 43	**.029**
Time	6.890	1, 43	**.012**
Group * Time	3.313	1, 43	***.076***

	Time 1 (Pre)	Time 2 (Post)	Paired *t*-test
Group	Mean (SD)	Mean (SD)	
MLD (*n* = 16)	.72 (.15)	.82 (.11)	*t*(15) = -2.803, ***p* =.013**
TD (*n* = 29)	.84 (.17)	.87 (.12)	*t*(28) = -1.070, *p* =.294

Supplementary Table 10. ANOVA and paired *t*-tests for changes in WJ-III Math Fluency in MLD and TD groups in the training group

	F	df_1, df_2	*p*
Omnibus ANOVA			
Group (MLD vs. TD)	31.810	1, 49	**<.001**
Time	1.074	1, 49	.305
Group * Time	7.268	1, 49	**.010**

	Time 1 (Pre)	Time 2 (Post)	Paired *t*-test
Group	Mean (SD)	Mean (SD)	
MLD (*n* =20)	84.50 (3.65)	90.40 (9.14)	*t*(19) = -3.183, ***p* =.005**
TD (*n* = 31)	103.34 (11.29)	101.90 (13.08)	*t*(30) = .817, *p* =.420

Supplementary References

Chang, H., Chen, L., Zhang, Y., Xie, Y., Adair, E., de los Angeles, C., Zanitti, G., Wassermann, D., Rosenberg-Lee, M., & Menon, V. (2022). Foundational number sense training gains are predicted by hippocampal – parietal circuits. *Journal of Neuroscience*, *42*, 4000-4015.

Dweck, C. S. (1999). *Self-Theories: Their Role in Motivation, Personality, and Development*. Psychol. Press.
