# Supplementary material for: Cognitive training enhances growth mindset in children through plasticity of cortico-striatal circuits
Source: NPJ Sci Learn. 2022 Nov 12;7:30. doi: 10.1038/s41539-022-00146-7 (PMC9653476; doi:10.1038/s41539-022-00146-7)
Supplement: Supplementary file 2 — Reporting Summary [file 41539_2022_146_MOESM2_ESM.pdf]

nature portfolio

Corresponding author(s): Lang Chen, Hyesang Chang, Vinod Menon

Last updated by author(s): Oct 4, 2022



# Reporting Summary

Nature Portfolio wishes to improve the reproducibility of the work that we publish. This form provides structure for consistency and transparency in reporting. For further information on Nature Portfolio policies, see our Editorial Policies and the Editorial Policy Checklist.

## Statistics

For all statistical analyses, confirm that the following items are present in the figure legend, table legend, main text, or Methods section.

| n/a | Confirmed | |
|---|---|---|
| ☐ | ☒ | The exact sample size ($n$) for each experimental group/condition, given as a discrete number and unit of measurement |
| ☐ | ☒ | A statement on whether measurements were taken from distinct samples or whether the same sample was measured repeatedly |
| ☐ | ☒ | The statistical test(s) used AND whether they are one- or two-sided<br>*Only common tests should be described solely by name; describe more complex techniques in the Methods section.* |
| ☐ | ☒ | A description of all covariates tested |
| ☐ | ☒ | A description of any assumptions or corrections, such as tests of normality and adjustment for multiple comparisons |
| ☐ | ☒ | A full description of the statistical parameters including central tendency (e.g. means) or other basic estimates (e.g. regression coefficient) AND variation (e.g. standard deviation) or associated estimates of uncertainty (e.g. confidence intervals) |
| ☐ | ☒ | For null hypothesis testing, the test statistic (e.g. $F$, $t$, $r$) with confidence intervals, effect sizes, degrees of freedom and $P$ value noted<br>*Give P values as exact values whenever suitable.* |
| ☒ | ☐ | For Bayesian analysis, information on the choice of priors and Markov chain Monte Carlo settings |
| ☒ | ☐ | For hierarchical and complex designs, identification of the appropriate level for tests and full reporting of outcomes |
| ☐ | ☒ | Estimates of effect sizes (e.g. Cohen's $d$, Pearson's $r$), indicating how they were calculated |

*Our web collection on statistics for biologists contains articles on many of the points above.*

## Software and code

Policy information about availability of computer code

| | |
|---|---|
| Data collection | No software was used for behavioral data collection. Brain imaging data were acquired on a 3T GE scanner. Numerical problem solving task was presented on E-Prime in the fMRI scanner. |
| Data analysis | Behavioral data were analyzed using R 3.6.1 and brain imaging data were preprocessed using SPM12 and analyzed using Matlab 9.2-9.8. |

For manuscripts utilizing custom algorithms or software that are central to the research but not yet described in published literature, software must be made available to editors and reviewers. We strongly encourage code deposition in a community repository (e.g. GitHub). See the Nature Portfolio guidelines for submitting code & software for further information.

## Data

Policy information about availability of data

All manuscripts must include a data availability statement. This statement should provide the following information, where applicable:

- Accession codes, unique identifiers, or web links for publicly available datasets
- A description of any restrictions on data availability
- For clinical datasets or third party data, please ensure that the statement adheres to our policy

Data used to support the findings of the current study are not openly available to ensure confidentiality of data acquired from participants and are available from corresponding authors upon reasonable request for research purposes within the agreement of the informed consent approved by the Institutional Review Board.

# Human research participants

Policy information about studies involving human research participants and Sex and Gender in Research.

| | |
|---|---|
| Reporting on sex and gender | We include both genders in our study. Data from 45 female (57%) and 34 male (43%) participants were analyzed in the current study. We report the distribution of gender in the two groups, training (29 females, 23 males) and control (16 females, 11 males), which was not significantly different (χ^2 = 0.003, p = .954). Sample size for each gender for each group is not sufficient to perform analysis of gender difference in behavioral and brain imaging data in the current study. Previous research suggests that there are no significant gender differences in early number skills (Kersey et al., 2018, npj Science of Learning). |
| Population characteristics | Seventy-nine children (45 females; age range = 6.76 - 10.02 years old, M = 8.20, SD = 0.65 at pre-visit). Participants were right-handed and had no history of psychiatric illness or neurological disorders. |
| Recruitment | Participants were recruited from multiple school districts in the San Francisco Bay Area. |
| Ethics oversight | Informed consent was obtained from the parent of each child and all study protocols were approved by the Stanford University Institutional Review Board. |

Note that full information on the approval of the study protocol must also be provided in the manuscript.

# Field-specific reporting

Please select the one below that is the best fit for your research. If you are not sure, read the appropriate sections before making your selection.

☒ Life sciences ☐ Behavioural & social sciences ☐ Ecological, evolutionary & environmental sciences

For a reference copy of the document with all sections, see nature.com/documents/nr-reporting-summary-flat.pdf

# Life sciences study design

All studies must disclose on these points even when the disclosure is negative.

| | |
|---|---|
| Sample size | The sample size for behavioral data analysis (52 children in the training group and 27 children in the control group) was considered sufficient (estimated power > 99 %) based on samples from previous cognitive training studies in children (range of Cohen's d: 1.1-1.3; Jolles et al., 2016, Cortex; Supekar et al., 2013, PNAS). For neuroimaging data analysis, 38 children in the training group were determined adequate (estimated power > 83%) based on previous fMRI studies of learning (range of Cohen's d: 1.0-1.2; Iuculano et al., 2015, Nat Comm, Supekar et al., 2021, Comm Bio). |
| Data exclusions | The exclusion criteria were established in previous studies, where the following participants were excluded:<br><br>Left-handed participants.<br>Participants with history of psychiatric illness or neurological disorders.<br>Participants with incomplete/invalid/missing behavioral or brain imaging data, low fMRI task accuracy (<40%), or poor quality brain imaging data or excessive head movement in either pre- or post-visit. |
| Replication | The current study did not test reproducibility of the findings. |
| Randomization | A two-group pretest-posttest design was employed in the current study. Group assignment (training, control) was not random, and baseline score was controlled in ANCOVA |
| Blinding | Blinding was not possible as the no-contact control group did not receive treatment. Inclusion of no-contact group allowed examination of effects of business-as-usual schooling experience |

# Reporting for specific materials, systems and methods

We require information from authors about some types of materials, experimental systems and methods used in many studies. Here, indicate whether each material, system or method listed is relevant to your study. If you are not sure if a list item applies to your research, read the appropriate section before selecting a response.

## Materials & experimental systems

| n/a | Involved in the study |
| --- | --- |
| ☒ | ☐ Antibodies |
| ☒ | ☐ Eukaryotic cell lines |
| ☒ | ☐ Palaeontology and archaeology |
| ☒ | ☐ Animals and other organisms |
| ☒ | ☐ Clinical data |
| ☒ | ☐ Dual use research of concern |

## Methods

| n/a | Involved in the study |
| --- | --- |
| ☒ | ☐ ChIP-seq |
| ☒ | ☐ Flow cytometry |
| ☐ | ☒ MRI-based neuroimaging |

# Magnetic resonance imaging

## Experimental design

| | |
| --- | --- |
| Design type | Event-related task fMRI |
| Design specifications | During both pre- and post-visits, children completed two runs of math problem-solving (addition) task (Figure 1a) in the MRI scanner. Given previously shown associations between growth mindset or positive attitude and math achievement (Blackwell et al., 2007, Child Development; Yeager et al., 2019, Nature; Chen et al., 2018, Psychol Sci), we chose to examine the neurobiological correlates of growth mindset during math problem solving. During each trial of our math problem-solving task, an addition problem was presented for 6 seconds, followed by a proposed solution which was presented for 1 second. Participants had a time window of up to 10.8 seconds (starting from the presentation of the solution) to indicate whether or not the proposed solution is correct. A total of 24 single-digit problems with operands from 2 to 9 (excluding ties) were presented in each run with the order of problems randomized across participants. Half of proposed solutions were valid, whereas the other half were invalid with solutions deviating from the correct answer by ±1 or ±2 units. The difficulty of each addition problem ("problem difficulty") was estimated by the correct answer to the problem, ranging from 5 to 17.<br><br>Since no control condition was included in the fMRI task, task-related brain responses were estimated with a parametric approach in the framework of general linear model implemented in SPM12. An event-related parametric fMRI task design allowed us to examine the effects of task difficulty, controlling for potential confounds such as visual perception and motor response. For each run at the individual level, problem difficulty of all addition problems was coded as a modulating parameter to examine the positive relationship between brain response and problem difficulty. Here, higher levels of brain responses indicated greater engagement during completion of more difficult math problems. |
| Behavioral performance measures | Accuracy and reaction times were collected for each participant. fMRI task runs with greater than 40% accuracy was included in data analysis to ensure attention to task and sufficient number of trials for analysis. |

## Acquisition

| | |
| --- | --- |
| Imaging type(s) | Functional MRI |
| Field strength | 3T |
| Sequence & imaging parameters | T2*-sensitive gradient echo spiral in/out pulse sequence, echo time (TE)=30ms, repetition time (TR)=2s, flip angle=80°, field-of-view=220mm, 31 axial slices parallel to the AC–PC, dimensions 3.4375 × 3.4375 × 4mm with 0.5-mm skip. |
| Area of acquisition | Whole brain scan |

Diffusion MRI ☐ Used ☒ Not used

## Preprocessing

| | |
| --- | --- |
| Preprocessing software | Data were analyzed using SPM12. The first five volumes were not analyzed to allow for signal equilibration. A linear shim correction was applied separately for each slice during reconstruction. Images were realigned to correct for motion and slice acquisition timing, spatially transformed to standard stereotaxic space (based on the Montreal Neurologic Institute coordinate system), and smoothed with a 6 mm full-width half-maximum Gaussian kernel to decrease spatial noise prior to statistical analysis. |
| Normalization | Data were normalized to standard stereotaxic space |
| Normalization template | MNI |
| Noise and artifact removal | Images were smoothed with a 6 mm full-width half-maximum Gaussian kernel to decrease spatial noise. |
| Volume censoring | To correct for deviant volumes resulting from spikes in movement, we used de-spiking procedures similar to those implemented in AFNI. Volumes with movement exceeding 0.5 voxels or spikes in global signal exceeding 5% were interpolated using adjacent scans. |

## Statistical modeling & inference

Model type and settings

A standard general linear model (GLM) was implemented in SPM12 to examine task-dependent brain responses associated with the parameter of problem difficulty (see "Design Specifications" above) for each fMRI run for each individual in each session. At the group level, we examined changes in brain activation (post-visit – pre-visit) associated with changes in growth mindset scores across individuals in the training group, using an F-test with contrast images of parametric estimates based on problem difficulty from individual statistics. In this F-test, we modeled time points (pre-visit, post-visit) as a within-subject factor and the difference score of growth mindset (post-visit – pre-visit) as a covariate. We examined both positive and negative contrasts of the interaction effects between time points and the difference score of growth mindset.

A general psychophysiological interaction (gPPI) method was used to examine task-dependent functional connectivity associated with the parameter of problem difficulty. Four ROIs were selected from regional activation analysis: the left and right dorsal ACC, right striatum (putamen), and right hippocampus. For each participant at each visit, a model was computed with the parameter of problem difficulty, each ROI time course, and their interaction term. The beta values of the interaction term were then extracted for all pairs of 4 ROIs (6 ROI-to-ROI links; Figure 3a) for each visit of each participant.

Effect(s) tested

Brain activity and functional connectivity

Specify type of analysis: ☐ Whole brain ☐ ROI-based ☒ Both

Anatomical location(s)

Anatomical locations were identified by the Harvard-Oxford atlas. For the cluster of dorsal ACC that crossed the two hemispheres, we used anatomical masks of the left and right cingulate gyri from the Automated Anatomical Labeling (AAL) atlas to identify local peaks in the left and right hemispheres separately. The nucleus accumbens (NAc) subdivisions were defined in the Brainnetome atlas.

Statistic type for inference
(See Eklund et al. 2016)

Cluster-wise multiple comparison correction ($p < .01$; spatial extent of 128 voxels based on Monte Carlo simulations)

Correction

Whole-brain analysis identified clusters of activation using a height threshold of $p < .01$, with family-wise error (FWE) corrections for multiple comparisons at the cluster level ($p < .01$; spatial extent of 128 voxels based on Monte Carlo simulations)

## Models & analysis

| n/a | Involved in the study |
|---|---|
| ☐ | ☒ Functional and/or effective connectivity |
| ☒ | ☐ Graph analysis |
| ☐ | ☒ Multivariate modeling or predictive analysis |

Functional and/or effective connectivity

See "Model types and settings" above

Multivariate modeling and predictive analysis

In a multivariate network regression analysis, we first examined whether changes in task-dependent functional connectivity (post-visit – pre-visit) between all 4 ROIs could predict changes in growth mindset scores (post-visit – pre-visit). Next, we examined associations between changes in each individual ROI-to-ROI link and changes in growth mindset scores. Pearson's correlation was used to examine associations between changes in functional connectivity for each ROI-ROI link and changes in growth mindset. Analyses were performed for both training and control groups to address training-specific brain plasticity associated with growth mindset gains. Finally, to assess the potential role of NAc in the ventral striatum in growth mindset, we additionally examined whether its functional connectivity with the 4 ROIs could jointly predict training-induced changes in growth mindset.
